# Antioxidant Activity of Bioactive Peptide Fractions from Germinated Soybeans Conjugated to Fe_3_O_4_ Nanoparticles by the Ugi Multicomponent Reaction

**DOI:** 10.3390/molecules26195726

**Published:** 2021-09-22

**Authors:** Yarelys Elena Augusto-Jimenez, Marcela González-Montoya, Dany Naranjo-Feliciano, Daniel Uribe-Ramírez, Eliseo Cristiani-Urbina, Carlos Díaz-Águila, Hernani Yee-Madeira, Rosalva Mora-Escobedo

**Affiliations:** 1 Instituto Politécnico Nacional–ESFM, U.P.A.L.M., San Pedro Zacatenco, Ciudad de Mexico 07738, Mexico; hernaniyee@gmail.com; 2 Instituto Politécnico Nacional–ENCB, U.P.A.L.M., San Pedro Zacatenco, Ciudad de Mexico 07738, Mexico; mrcela_mecs@hotmail.com (M.G.-M.); daniel.uriberam@gmail.com (D.U.-R.); ecristiani@ipn.mx (E.C.-U.); 3 Centro Nacional de Sanidad Agropecuaria, San Jose de las Lajas 32700, Mayabeque, Cuba; danynaranjofeliciano@gmail.com; 4 Centro de Biomateriales, Universidad de La Habana, Plaza de la Revolucion 10400, La Habana, Cuba; cdiaz@biomat.uh.cu

**Keywords:** soybean, peptides, antioxidant capacity, magnetite nanoparticles, conjugation, multicomponent reaction

## Abstract

The conjugation of biomolecules to magnetic nanoparticles has emerged as promising approach in biomedicine as the treatment of several diseases, such as cancer. In this study, conjugation of bioactive peptide fractions from germinated soybeans to magnetite nanoparticles was achieved. Different fractions of germinated soybean peptides (>10 kDa and 5–10 kDa) were for the first time conjugated to previously coated magnetite nanoparticles (with 3-aminopropyltriethoxysilane (APTES) and sodium citrate) by the Ugi four-component reaction. The crystallinity of the nanoparticles was corroborated by X-ray diffraction, while the particle size was determined by scanning transmission electron microscopy. The analyses were carried out using infrared and ultraviolet–visible spectroscopy, dynamic light scattering, and thermogravimetry, which confirmed the coating and functionalization of the magnetite nanoparticles and conjugation of different peptide fractions on their surfaces. The antioxidant activity of the conjugates was determined by the reducing power and hydroxyl radical scavenging activity. The nanoparticles synthesized represent promising materials, as they have found applications in bionanotechnology for enhanced treatment of diseases, such as cancer, due to a higher antioxidant capacity than that of fractions without conjugation. The highest antioxidant capacity was observed for a >10 kDa peptide fraction conjugated to the magnetite nanoparticles coated with APTES.

## 1. Introduction

The main challenges for magnetic nanoparticles (MNPs) used in biomedicine are related to their tendency to agglomerate and lack of biocompatibility [1]. Regarding biocompatibility, iron oxide nanoparticles (NPs) maghemite (γ-Fe_2_O_3_) and magnetite (Fe_3_O_4_) are advantageous for in vivo applications. Unlike other materials with good magnetic behavior, iron cell homeostasis is well-controlled by absorption, excretion, and storage processes. Even excess iron is efficiently removed from the body [2]. Specially, different works show that the superparamagnetic behavior of magnetite NPs is closely related to their nanometric size [3]. This property is essential for the in vivo applications of the material as it ensures that no magnetization remains in the system after stopping the action of an external magnetic field. Moreover, uncoated magnetite NPs tend to decrease the surface free energy by forming stable aggregates under physiological conditions [4]. In this regard, it has been observed that the coupling of different types of biomolecules to Fe_3_O_4_ NPs increases the stability of the system while directing it towards the desired biological target. Thereby, magnetite has been coupled to polymers and different anticancer drugs, with prominent applications in biomedicine, nanoscience, and nanotechnology [5,6,7]. These conjugates are of interest mainly because the benefits of the Fe_3_O_4_ NPs and biomolecules are combined in the same system.

Peptide–Fe_3_O_4_ conjugates are promising for biotechnological applications [8,9,10], particularly because peptides from natural sources and synthetic derivatives have been identified as promising for the treatment of diseases, such as cancer [11,12]. From the conjugation of peptides to Fe_3_O_4_ NPs, one could not only selectively transport drugs towards biological targets, but also treat different diseases using magnetic hyperthermia [13,14,15,16]. Despite the advances in the development of methodologies for the conjugation of peptides to magnetite NPs, the current protocols are still limited by long and complex reaction steps. In most cases, they are based on classical peptide coupling between peptides and MNPs functionalized with carboxylic acid groups [17]. In some studies, surface modification of MNPs has been performed with ligands suitable for their subsequent coupling to peptides through click chemistry [14]. This restricts and makes the application of peptide–Fe_3_O_4_ conjugates in biomedical investigations more expensive.

Several procedures for the combination of complex molecules based on multicomponent reactions have been reported [18,19]. Multicomponent protocols have a high potential owing to their high chemical efficiency, simple execution, and structural diversity at low synthesis costs [20]. One of the most used is the Ugi four-component (U-4C) reaction, in which the condensation of an oxo derivative, primary amine, carboxylic acid, and isonitrile leads to the formation of a dipeptoid in a single reaction step. The N-substituted amide in peptoids derived from the U-4C reaction is a structural motif of biological relevance because it prevents proteolytic degradation and limits the conformational orientation of peptide structures [21]. However, to the best of our knowledge, this type of reaction has not been used for the conjugation of bioactive peptides to MNPs.

On the other hand, several studies demonstrated the important role of oxidative stress and presence of different reactive oxygen and nitrogen species (ROS and RNS, respectively) in numerous noncommunicable diseases, such as cardiovascular diseases, diabetes, atherosclerosis, arthritis, and cancer [22]. These diseases lead to approximately 41 million deaths per year, equivalent to 71% of all deaths globally [23]. Some peptides and biomimetic peptides inhibit the generation of ROS and other free radicals [24,25]. Therefore, the search for antioxidant peptide structures that can prevent oxidative stress and its associated adverse effects is of increasing interest. In this sense, González-Montoya et al. evaluated the anti-inflammatory, antioxidant, and antiproliferative activities of three peptide fractions obtained from germinated soybeans [26,27]. These peptide fractions exhibited exceptional antioxidant and antiproliferative activities in breast and cervical cancer cell lines. Motivated by these studies, we present the synthesis, characterization, and biological evaluation of conjugates of Fe_3_O_4_ NPs with bioactive peptide fractions from germinated soybeans. To the best of our knowledge, these bioconjugates are reported for the first time. A multicomponent procedure is also used for the first time in the conjugation of bioactive peptide fractions from germinated soybeans to magnetite NPs. 

## 2. Results and Discussion 

**Scheme 1:**
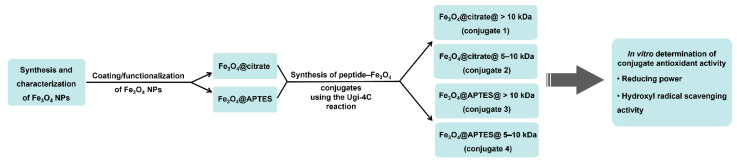
Experimental workflow.

A procedure for the conjugation of bioactive peptide fractions to magnetite NPs was developed (Scheme 1). The methodology comprised three fundamental parts: (1) synthesis of Fe_3_O_4_ NPs, (2) functionalization of Fe_3_O_4_ NPs in the form of either carboxylic acid or amine, and (3) conjugation using the U-4C reaction of the peptide fractions extracted from germinated soybeans to the functionalized Fe_3_O_4_ NPs. Once the desired conjugates were obtained, the antioxidant activity of both the peptide–Fe_3_O_4_ conjugates as well as the peptide fractions and coated magnetite were determined.

### 2.1. Characterization of Fe_3_O_4_

**Figure 1:**
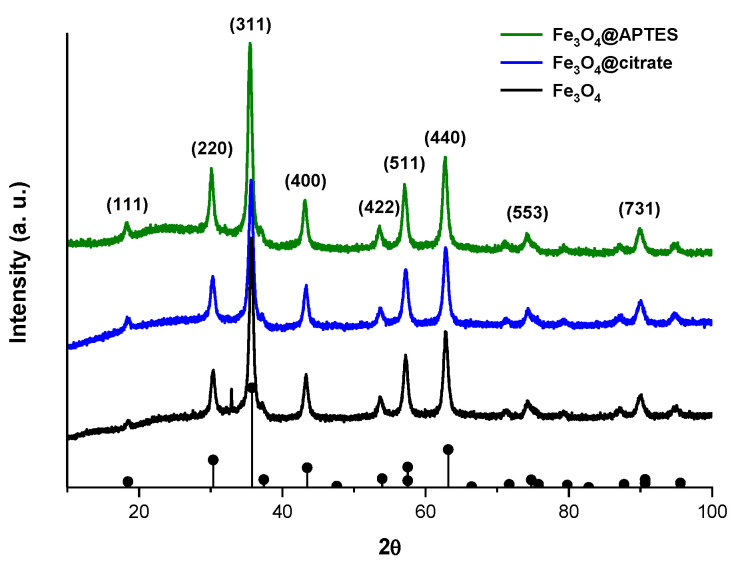
XRD patterns of the uncoated magnetite (Fe_3_O_4_) and magnetite samples coated with sodium citrate (Fe_3_O_4_@citrate) and APTES (Fe_3_O_4_@APTES).

The X-ray diffraction (XRD) pattern of the synthesized magnetite is shown in Figure 1 (black profile). The main peaks associated with the characteristic spinel structure of magnetite were observed at 18.3, 30.2, 35.6, 43.3, 53.6, 57.3, 62.9, 74.5, and 90.6°. These peaks are indexed to the (111), (220), (311), (400), (422), (511), (440), (533), and (731) hkl planes of the Joint Committee on Powder Diffraction Standards (JCPDS) 19629 magnetite standard pattern, respectively. The estimated Fe_3_O_4_ cell parameter was 8.3824 Å and the average crystallite size calculated using the Debye–Scherrer equation (Equation (1)) was 17 nm. This last value allowed confirming the nanometric size of the obtained magnetite.

**Figure 2:**
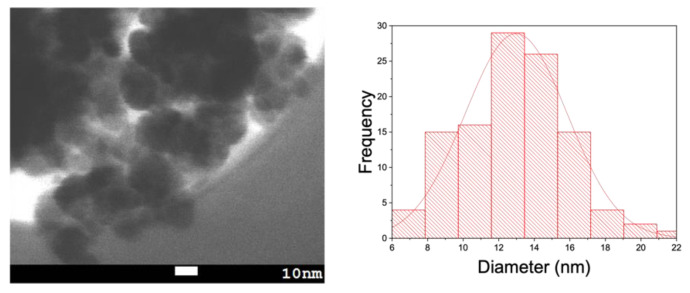
Microscopy image of uncoated Fe_3_O_4_ with the corresponding histogram.

Additional information about the size and morphology of Fe_3_O_4_ NPs was obtained using scanning transmission electron microscopy (STEM) (Figure 2). The particle size determined was 13 ± 5 nm. The fact that this value is similar to the calculated crystallite size suggests that the system was monocrystalline. In the STEM images, it is observed that uncoated Fe_3_O_4_ NPs have spherical morphology and are agglomerated, most likely because of their small sizes.

**Figure 3:**
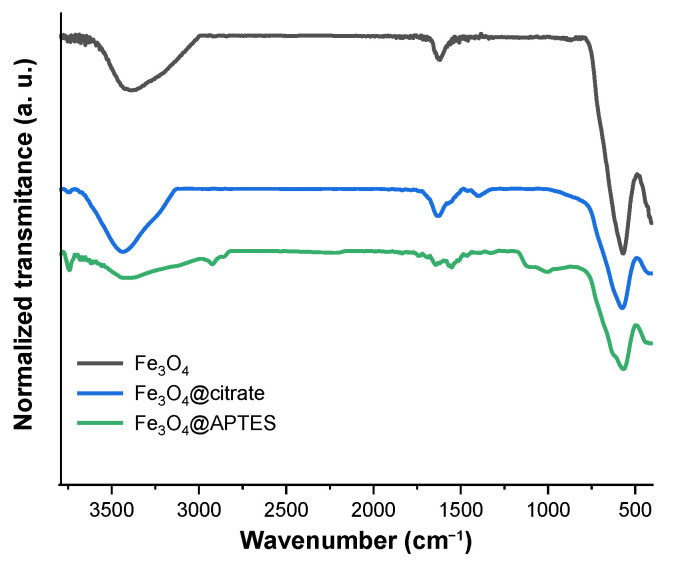
IR spectra of the uncoated magnetite (Fe_3_O_4_) and magnetite samples coated with sodium citrate (Fe_3_O_4_@citrate) and APTES (Fe_3_O_4_@APTES).

The synthesis of Fe_3_O_4_ was also corroborated by infrared (IR) spectroscopy (Figure 3, black spectrum). From the assignment of the absorption bands observed in the IR spectra obtained, it was possible to analyze the respective functional groups of the molecules present in each of the synthesized materials. Thus, a broad band centered at 3252 cm^−1^, corresponding to the overlapping valence vibrations of the N–H and O–H bonds, was observed. It was assigned to NH_4_^+^ and OH^−^ ions and adsorbed water molecules on the surface of MNPs. In addition, the band at 1628 cm^−1^ can be attributed to the bending of the NH_4_^+^ group on the magnetite surface. Finally, at 573 cm^−1^, a band assigned to the valence vibration of the Fe–O bond of Fe_3_O_4_ was observed. 

Finally, the hydrodynamic diameter of Fe_3_O_4_ was determined using dynamic light scattering (DLS) measurements. The value obtained (180 ± 70 nm) was used as a reference for comparison to the coated magnetite samples.

### 2.2. Characterization of the Functionalized Fe_3_O_4_

As mentioned in the Introduction, though magnetite is a biocompatible material, its stabilization in a physiological environment is only possible after coating [2,4]. In addition, functionalization enables the conjugation with therapeutic biomolecules and contributes to achieving effective directionality of magnetite nanoparticles towards the site of interest inside the body [7]. In this work, it was decided to carry out the coating/functionalization of the Fe_3_O_4_ NPs in a single step. Thus, from the use of sodium citrate and 3-aminopropyltriethoxysilane (APTES), it was possible to functionalize the magnetite surface in the form of carboxylic acid and amine, respectively. Both functional groups are required for subsequent conjugation to the peptide fractions from the Ugi 4C reaction. Each of the variants made is described below.

#### 2.2.1. Characterization of Fe_3_O_4_@citrate

Carboxylate is one of the functional groups that most strongly binds to the surface of magnetite NPs [28]. Sodium citrate (or the corresponding acid), a small biocompatible molecule, has three carboxylate groups in its structure. Only one or two of the carboxylate functions of sodium citrate have been found to coordinate to the magnetite surface, allowing at least one of them to be exposed to the medium [29]. This effect is very convenient for the application of the magnetic system since it protects the nanoparticulate nucleus from the possible formation of aggregates while providing it with hydrophilicity and functionalizing it for subsequent derivatizations and couplings. Here, after the synthesis of Fe_3_O_4_ NPs coated with sodium citrate (Fe_3_O_4_@citrate), the resulting product was characterized.

**Figure 4:**
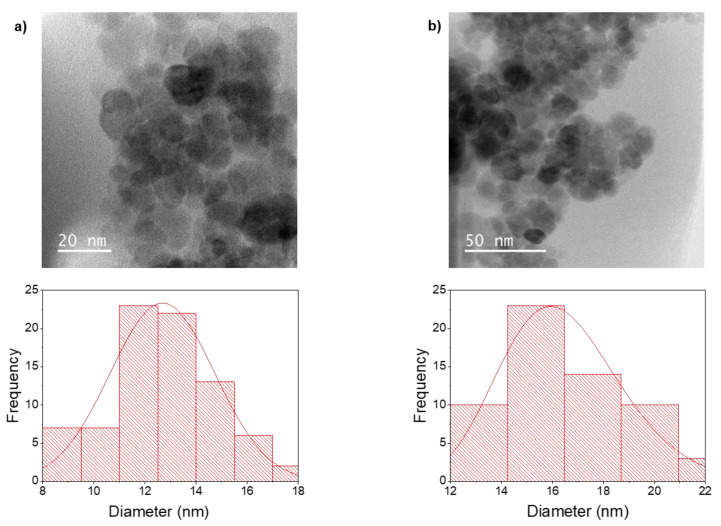
Microscopy images of (**a**) Fe_3_O_4_@citrate and (**b**) Fe_3_O_4_@APTES with the corresponding histograms.

The Fourier-transform infrared (FTIR) spectroscopy analysis of the MNPs coated with sodium citrate suggested that the desired product was obtained (Figure 3, blue spectrum). The three bands at 3435, 1629, and 1398 cm^−1^ indicate carboxylate in the sample. These signals correspond to the valence vibrations of the O–H, C=O, and COO– bonds, respectively. The band corresponding to Fe_3_O_4_ remained at 537 cm^−1^. In addition, the XRD patterns of the starting magnetite (Figure 1, black profile) and that coated with citrate (Figure 1, blue profile) were similar, which suggests that the crystallinity of the material was not affected by the coating. However, the calculated cell parameter was 8.3643 Å. The difference with respect to the cell parameter of the uncoated magnetite indicates a strong interaction between citrate atoms and magnetite, which results in the contraction of the unit cell of the starting material [30]. The average crystallite size determined using the Debye–Scherrer equation (Equation (1)) was 16 nm. This value is similar to the particle size estimated using STEM (13 ± 3 nm). The similarity between these results suggests that the compound was monocrystalline. Figure 4a shows a microscopy image and obtained size distribution with the corresponding histogram. The obtained NPs had spherical morphology with some aggregation because of their small sizes.

Another evidence of the presence of citrate on the surface of the MNPs is the increase in the hydrodynamic diameter (284 ± 140 nm) relative to that of the uncoated MNPs. As some of the polar groups of citrates (carboxylate and hydroxyl) are dangling towards the surrounding medium, a larger hydration layer is generated. Finally, the weight percentage of the coating material on magnetite, with respect to the uncoated Fe_3_O_4_, was determined using thermogravimetric analysis (TGA). The weight loss was 2.21%, confirming that magnetite was coated with an organic material.

#### 2.2.2. Characterization of Fe_3_O_4_@APTES

The functionalization of the Fe_3_O_4_ NPs surface in the amine form was carried out from a silylation reaction with the use of APTES. This molecule is the most widely used alkoxysilane for coating magnetite NPs since it provides the highest density of amine groups on the surface of the magnetic core [31].

The IR spectrum of Fe_3_O_4_@APTES showed characteristic bands that confirmed the presence of the expected APTES coating (Figure 3, green spectrum). Thus, a wide band, centered at 3399 cm^−1^, was observed, attributed to the overlapping valence vibrations of the O–H and N–H bonds of the hydroxyl and amine groups in APTES. The bands corresponding to the antisymmetric and symmetric vibrations of the APTES methylene groups appeared at 2928 and 2842 cm^−1^, respectively. The bands corresponding to the vibrations antisymmetric and symmetric of the NH_2_ group in APTES were observed around 1642 and 1549 cm^−1^, respectively. The presence of silanol groups (Si–OH) and siloxanes (Si–O–Si) on the surface of the MNPs was verified by the bands of the valence vibrations of the Si–O–Si and Si–OH bonds at 1396 and 1015 cm^−1^, respectively. Finally, the Fe_3_O_4_ band was maintained at 573 cm^−1^, suggesting the presence of magnetite in the conjugate. Moreover, no differences were observed between the XRD patterns of the coated and uncoated magnetite samples (Figure 1, green and black profile, respectively), which confirms that the crystallinity of the material was not affected by the coating with APTES. 

The calculated cell parameter of Fe_3_O_4_@APTES was 8.3744 Å, slightly lower than that determined for the uncoated magnetite, as the Fe and O atoms are closer to each other in the unit cell owing to the APTES molecules [30]. The crystallite size of the APTES-coated NPs was 17 nm, which confirms that the compound maintained its nanoscale dimensions. This value is very similar to the particle size obtained using STEM (16 ± 3 nm), which suggests that the system was monocrystalline. The STEM images (Figure 4b) show that the coated NPs have spherical morphology and are agglomerated because of their small sizes. The weight percentage of the APTES-coated Fe_3_O_4_ was determined using TGA. A weight loss of 5.22% with respect to the uncoated Fe_3_O_4_ was obtained, which confirmed the coating on the magnetite surface.

### 2.3. Determination of the Degree of Substitution of Functionalized MNPs

The degree of substitution on the magnetic oxide was assessed, i.e., the amount of free functional groups on the surface of Fe_3_O_4_. For this purpose, the adsorption capacity of Cu (II) on the functionalized MNPs was determined using a spectrophotometric analysis. The results are presented in Table 1. To assess the molar quantity of free carboxylate groups on the MNPs coated with sodium citrate, it was considered that 2 M of carboxylate is necessary to complex 1 M of Cu^2+^. In the case of MNPs coated with APTES, it was assumed that the Cu^2+^ ions would form a complex with tetrahedral geometry with free amine groups on the magnetite [32]. Using this procedure, approximate molar quantities of free carboxylates or amine groups per gram of Fe_3_O_4_ were obtained.

The calculations for the subsequent synthesis step were carried out using the average value of the loading of functional groups on the Fe_3_O_4_ surface.

### 2.4. Characterization of Peptide–Fe_3_O_4_ Conjugates

**Figure 5:**
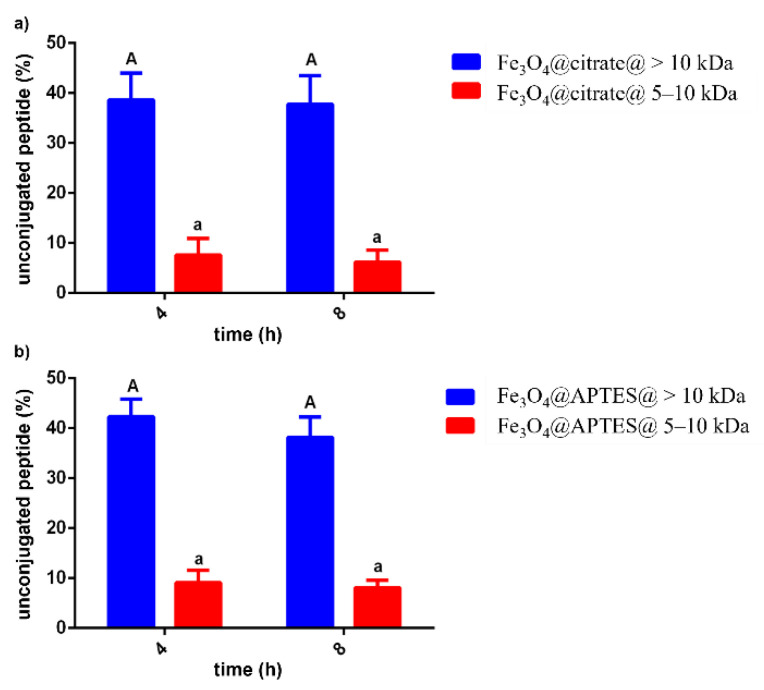
Percentages of unconjugated peptides to coated Fe_3_O_4_: (**a**) Fe_3_O_4_@citrate@ > 10 kDa, Fe_3_O_4_@citrate@ > 5–10 kDa; (**b**) Fe_3_O_4_@APTES@ 10 kDa, Fe_3_O_4_@APTES@ 5–10 kDa. The determinations were carried out in triplicate. The results are expressed as the means ± standard deviation. Different letters indicate statistical difference between the samples for each reaction time (*p* ≤ 0.05).

The main objective of this study was to develop a simple procedure for the conjugation of bioactive peptide fractions extracted from germinated soybeans to magnetite NPs. To this end, the U-4C reaction was chosen because it occurs in a single step and can be easily implemented [33,34]. The results for the remaining peptide concentrations are shown in Figure 5. There were no statistically significant differences in the percentage of free peptides at 4 and 8 h in either variant for the same peptide fraction. This suggests that the reaction ended after 4 h. However, the difference between the percentages of conjugation when the fraction >10 kDa was used (approximately 60%) and that at 5–10 kDa (approximately 92%) was remarkable. This was expected considering the smaller size of the peptides contained in the latter, which would facilitate the multicomponent process. On the other hand, the yields of the developed conjugation process can be considered good in all the cases, considering that the magnetic component of the reaction is solid-support-dispersed, while the other reactants are dissolved in the medium [35].

**Figure 6:**
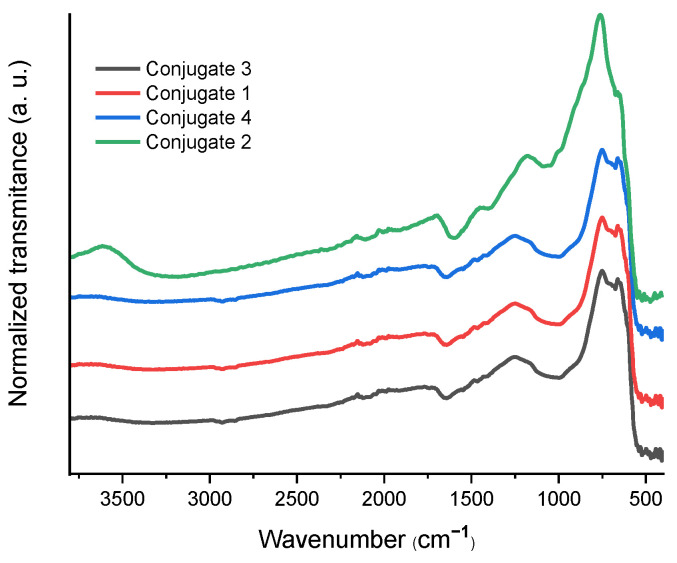
FTIR spectra of the final conjugates: Fe_3_O_4_@citrate@ > 10 kDa (conjugate 1), Fe_3_O_4_@citrate@ 5–10 kDa (conjugate 2), Fe_3_O_4_@APTES@ > 10 kDa (conjugate 3), and Fe_3_O_4_@APTES@ 5–10 kDa (conjugate 4).

The conjugates were characterized using FTIR spectroscopy (Figure 6). In all the cases, the bands that confirmed the presence of carbonyl groups in each of the structures on the MNPs were observed at approximately 1620 cm^−1^. In addition, the band at 573 cm^−1^ was retained, which corroborated the presence of Fe_3_O_4_.

### 2.5. Antioxidant Activities of the Conjugates

**Figure 7:**
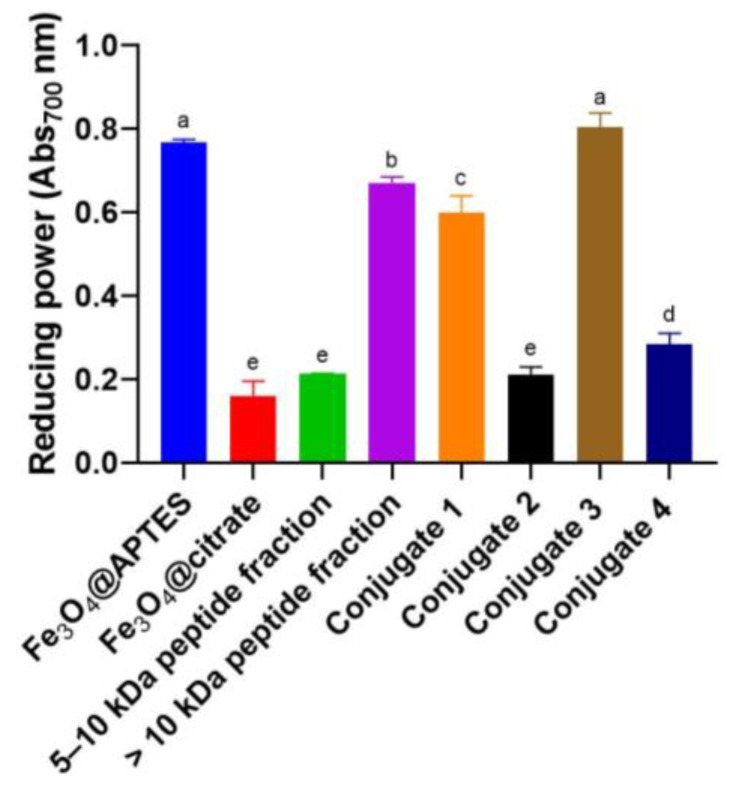
Average results of the RPs of coated magnetite (Fe_3_O_4_@APTES and Fe_3_O_4_@citrate) and of the conjugates and nonconjugated peptide fractions at 15 mg/mL: Fe_3_O_4_@citrate@ > 10 kDa (conjugate 1), Fe_3_O_4_@citrate@ 5–10 kDa (conjugate 2), Fe_3_O_4_@APTES@ > 10 kDa (conjugate 3), Fe_3_O_4_@APTES@ 5–10 kDa (conjugate 4), and nonconjugated peptide fractions (>10 kDa and 5–10 kDa peptide fractions). The determinations were carried out in triplicate. The results are expressed as the means ± standard deviation. Different letters indicate statistical difference between the samples (*p* ≤ 0.05).

**Figure 8:**
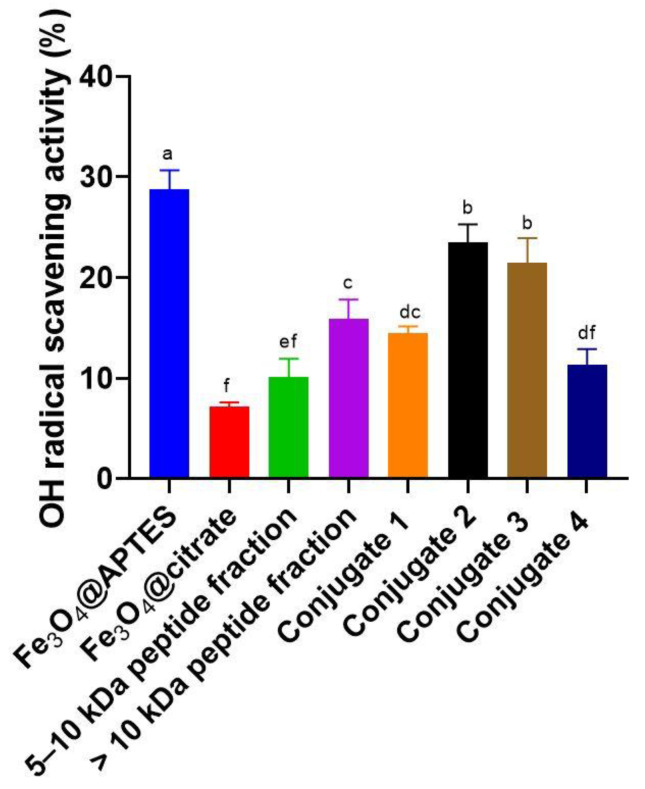
Average results of the OH· scavenging activities of coated magnetite (Fe_3_O_4_@APTES and Fe_3_O_4_@citrate) and of the conjugates and nonconjugated peptide fractions at 15 mg/mL: Fe_3_O_4_@citrate@ > 10 kDa (conjugate 1), Fe_3_O_4_@citrate@ 5–10 kDa (conjugate 2), Fe_3_O_4_@APTES@ > 10 kDa (conjugate 3), Fe_3_O_4_@APTES@ 5–10 kDa (conjugate 4), and nonconjugated peptide fractions (>10 kDa and 5–10 kDa peptide fractions). The determinations were carried out in triplicate. The results are expressed as the means ± standard deviation. Different letters indicate statistical differences between the samples (*p* ≤ 0.05).

The association between the generation of ROS and the development of chronic degenerative diseases has been reported [27]. Furthermore, in cellular metabolism, oxidative compounds are produced constantly, so it is important to counteract these oxidizing spices to maintain a balance of intra- and extracellular homeostasis. Hence, the antioxidant capacities of the conjugates obtained in this study were evaluated. The determinations were made by verifying their reducing power (RP) and hydroxyl radical (OH·) scavenger abilities. The results presented below (Figure 7 and Figure 8) correspond to the tests carried out at a concentration of 15 mg/mL, at which a stronger effect was observed in the determinations. All the results were compared to those obtained for the nonconjugated peptide fractions and for the coated magnetite (Fe_3_O_4_@APTES and Fe_3_O_4_@citrate).

The RPs of the coated magnetite, as well as those of the conjugates and nonconjugated peptide fractions (Figure 7), were expressed as absorbances, where 1 represents the highest value. The RP of a sample refers to its ability to act as a proton acceptor (or electron donor) in an oxidation–reduction reaction and, therefore, the number of basic groups in the conjugates could be related to the obtained RP. In this sense, Fe_3_O_4_@APTES, with free amine groups on the magnetite surface, showed the highest RP value. Regarding the conjugates, those in which magnetite was conjugated to the fractions containing the peptides with masses >10 kDa (conjugates 1 and 3) had the highest RP values, with statistically significant differences between them and the conjugates 2 and 4. This result agrees with the study by González-Montoya et al. [27], who observed that the >10 kDa (RP = 0.7) fraction had a higher RP than the 5–10 kDa fraction (RP = 0.3). This could be attributed to the higher amount of basic amino acids in the >10 kDa fraction. A synergistic effect was observed when conjugating the peptide fractions to Fe_3_O_4_@APTES (conjugate 3 with respect to the >10 kDa peptide fraction), surely, due to the contribution of the amine groups that remained free, unreacted during the multicomponent reaction, on the surface of the magnetite coated with the APTES. It was also observed that the conjugation of the >10 kDa peptide fraction to magnetite NPs coated with sodium citrate (conjugate 1) slightly decreased the RP with respect to that of the peptide fraction, whilst the RP of the 5–10 kDa peptide fraction conjugated to Fe_3_O_4_@citrate (conjugate 2) did not exhibit statistically significant differences with respect to the nonconjugated fraction. In this way, conjugate 3 represents an opportunity for future evaluations of biological activities related with oxidative stress in chronic diseases.

The hydroxyl radical (OH·) is one of the most reactive free radicals and is generated by the Fenton reaction in cells [27]. This radical can be transformed into a superoxide anion and hydrogen peroxide in the presence of metal ions such as copper and iron. The OH· scavenging activities of the coated magnetite (Fe_3_O_4_@APTES and Fe_3_O_4_@citrate), as well as those of the conjugates and nonconjugated peptide fractions, are shown in Figure 8. According to the data, the nature of the groups exposed to the medium on each sample determines the properties of the compound. In the case of conjugates with the fraction > 10 kDa (conjugates 1 and 3), the activity is higher when it is used as an acidic component of the multicomponent reaction (conjugate 3). Therefore, in the final compound, the peptides would mostly expose their amine groups (which are apparently involved in the measured activity) to the environment. In the conjugates with the 5–10 kDa fraction (conjugates 2 and 4), the opposite effect was observed. The peptides contained in this fraction exerted a stronger OH· scavenging effect by exposing their acidic groups (conjugate 2). Remarkably, the OH· scavenging activities of conjugates 2 and 3 were significantly higher than that of the nonconjugated fractions. This result could be due to a synergistic effect of the respective carboxylic acid (from Fe_3_O_4_@citrate) and amine (from Fe_3_O_4_@APTES) groups in the corresponding final conjugates.

These results suggest that conjugates 2 and 3 could be a used as antioxidant conjugates and evaluated in the future in chronic diseases related with oxidative stress, such as cancer since it has been reported that antioxidant activity can modulate the activity of key proteins involved in the control of cell cycle progression and may influence the expression of many associated genes [36].

## 3. Materials and Methods 

### 3.1. Materials

Ferrous chloride tetrahydrate (FeCl_2_·4H_2_O, PA), sodium citrate dihydrate (Na_3_C_6_H_5_O_7_·2H_2_O, ACS >99%), APTES (97%), paraformaldehyde, 1-pentynylisonitrile, trichloroacetic acid (TCA), phenanthroline, and ferrous sulphate (FeSO_4_) were purchased from Sigma-Aldrich, Mexico City, Mexico. Ferric chloride hexahydrate (FeCl_3_·6H_2_O, 99.0%) and the ammonium hydroxide solution (NH_4_OH, 28.4%) were purchased from J.T. Baker, Mexico City, Mexico. Hydrochloric acid (HCl, PA) and potassium ferricyanide (K_3_[Fe(CN)_6_]) were purchased from Fermont, Monterrey, Mexico, and hydrogen peroxide (H_2_O_2_) from Reasol, Mexico city, Mexico. The peptide fractions were supplied by Laboratorio de Bioquímica de la Nutrición (ENCB-IPN), Mexico City, Mexico.

### 3.2. Synthesis of Fe_3_O_4_ NPs

The NPs were synthesized using the coprecipitation method [37]. A mixture of acidic solutions (HCl, 2 M) of the FeCl_2_·2H_2_O (5 mL, 5 M) and FeCl_3_·6H_2_O (20 mL, 2.5 M) salts was added to an aqueous solution of NH_3_ (250 mL, 0.7 M) contained in a three-neck balloon using a peristaltic pump. All the solutions were bubbled with N_2_ (g) before the synthesis. The reaction mixture was kept at room temperature under an N_2_ (g) atmosphere under mechanical stirring (500 rpm) and ultrasound-treated for 25 min. After the reaction time, the obtained MNPs were separated using a neodymium magnet. Subsequently, they were washed with 100 mL of deionized water and separated again to obtain a black powder corresponding to Fe_3_O_4_, which was dried in a desiccator containing activated silica for 12 h.

### 3.3. Coating/Functionalization of Fe_3_O_4_ NPs

#### 3.3.1. Fe_3_O_4_@citrate

The functionalization of the MNPs as carboxylic acids was carried out by coating Fe_3_O_4_ with sodium citrate. Fe_3_O_4_ (500 mg) was added to an aqueous solution of Na_3_C_6_H_5_O_7_·2H_2_O (50 mL, 1 mM). The reaction mixture was mechanically stirred for 4 h at room temperature, and then adjusted to pH = 9 using an aqueous solution of NH_3_ (0.7 M). The obtained precipitate was magnetically separated and washed twice with 50 mL of deionized water. Finally, it was dried (as explained for the synthesis of the Fe_3_O_4_ NPs), and Fe_3_O_4_@citrate was obtained in the form of a black powder.

#### 3.3.2. Fe_3_O_4_@APTES

Additionally, the functionalization of the MNPs as amines was carried out by modifying the surface of Fe_3_O_4_ with APTES [31]. Briefly, 500 mg of Fe_3_O_4_ were redispersed in a mixture of ethanol (80 mL) and deionized water (40 mL). The dispersion was adjusted to pH = 10 with an aqueous solution of NH_3_ (0.7 M) and homogenized in an ultrasonic bath. Subsequently, an alcoholic solution of APTES (5 mL, 10%) was dripped to the reaction mixture under constant stirring. After 24 h, the precipitate was separated with a neodymium magnet and washed twice with 50 mL of ethanol. Finally, the product was dried according to the procedure described above to obtain Fe_3_O_4_@APTES in the form of a black powder.

### 3.4. Cu(II) Adsorption Capacity of Functionalized NPs

Once the functionalization of MNPs was completed, the degree of substitution on the magnetic oxide was calculated. The determination could be carried out using the bicinchoninate method from the quantification of Cu(II) ions remaining in aqueous dispersions [38]. An aqueous solution of CuSO_4_·5H_2_O (35.5 mL, 100 mg/L) was prepared in an Erlenmeyer flask. Subsequently, 5 mL of the solution were collected, and a bicinchoninic acid kit was added. The absorbance was measured using an ultraviolet (UV)–visible (Vis) spectrophotometer at 560 nm (time: 0 h), and then the concentration was determined. The functionalized MNP sample (0.03 g) was added to the remaining solution (30.5 mL) and the pH was adjusted to 5 with HCl (0.1 M). The reaction mixture was mechanically stirred with a glass-lined magnetic stirrer at 140 rpm for 1 h. The magnetic system was then separated with a neodymium magnet. The supernatant (0.25 mL) was then collected, filtered, and diluted with 4.75 mL of distilled water, and a bicinchoninic acid kit was added. The absorbance was measured at 560 nm (time: 1 h), and then the concentration was determined. The last absorbance measurement was performed 24 h after the functionalized MNPs were in contact with CuSO_4_·5H_2_O (time: 24 h). Each measurement was performed in duplicate. 

### 3.5. Synthesis of Peptide–Fe_3_O_4_ Conjugates by the U-4C Reaction

To conjugate the peptide fractions to Fe_3_O_4_ using the U-4C reaction, the amino component (the peptide fractions or Fe_3_O_4_@APTES) and four equivalents (eq) of paraformaldehyde were reacted at room temperature for 24 h in the smallest required volume of phosphate-buffered saline (PBS) (pH = 7.4). The acid component (Fe_3_O_4_@citrate or peptide fractions) was then added; 10 min later, 1-pentynylisonitrile (4 eq) was added. The reactions were monitored at intervals of 4 h over 8 h and carried out in quintuplicate. Finally, the products were washed twice with 1 mL of distilled water and dried, as stated for the synthesis of Fe_3_O_4_ NPs.

### 3.6. Extension of the Conjugation

The amount of peptide remaining after conjugation to MNPs by the U-4C reaction was determined according to the Bradford assay [39]. The supernatants of each reaction (10 μL) were deposited with 100 μL of the Bradford reagent in a 96-well microplate. The readings were carried out at a wavelength of 595 nm. The remaining peptide concentration values were determined using a calibration curve of bovine serum albumin (BSA). 

### 3.7. NP Characterizations Using X-ray Diffraction, Fourier-Transform Infrared Spectroscopy, Scanning Transmission Electron Microscopy, Thermogravimetric Analysis, Dynamic Light Scattering, and Spectrophotometric Analysis 

XRD measurements were performed using a Bruker D8 Advance diffractometer. Cu *Kα* (*λ* = 1.54183 Å) incident radiation was used. The measurements were carried out in the range of 5–100° with an increment of 0.02° and scan speed of 10 s. The cell parameters were calculated using the Powdercell 2.4 software. The average crystallite size was determined using the Debye–Scherrer equation (Equation (1))

(1) $$D=\;\frac{\kappa \lambda }{\beta \cos\theta }.$$

where *D* is the crystallite size, *κ* is the Debye–Scherrer constant (0.89), *λ* is the operation wavelength, *β* is the average width of the most intense peak, and *θ* is the Bragg angle.

FTIR spectra were recorded using an Equinox 55 Bruker spectrometer in the absorbance measurement range of 4000 to 400 cm^−1^. TGA measurements were performed using a Netzsch STA 409 PC Luxx thermal analyzer coupled to the FTIR equipment. Approximately 10 mg of the sample were heated from 25 to 1000 °C and analyzed at the heating flux of 20 °C/min in an Ar atmosphere. DLS measurements were carried out using a Zetasizer Nano sampler (Malvern Instruments, Cambridge, UK) equipped with a He–Ne laser (λ = 633 nm). The measurements were carried out in square polystyrene cells at 25 °C in the range of 0.3–10 μm using H_2_O as a dispersion medium. The absorbance performance was assessed using two different types of equipment. To determine the adsorption capacity of Cu^2+^ ions, the absorbance values were obtained at 560 nm using a Genesys 10 UV spectrophotometer (Thermo Electron Corporation, Madison, WI, USA). During the experiment with the peptide fractions, the absorbances were measured using a microplate reader (Thermo Scientific Multiskan GO, Waltham, MA, USA). The readings were carried out at 536, 595, and 700 nm depending on the system and properties to be determined. The morphology and average size of the NPs were analyzed using STEM images. The measurements were carried out using a JEM-ARM200CF electronic microscope with the resolution of 80 pm for the employed mode. The acceleration voltage was 200 keV. The analysis of the STEM images to determine the NP average size was carried out with the ImageJ (Origin(Pro) Corporation, Northampton, MS, USA), and the histograms were obtained using Origin v9.0, (OriginLab Corporation, Northampton, MS, USA).

### 3.8. In Vitro Determination of the Antioxidant Activity of the Samples

#### 3.8.1. Reducing Power (RP)

The RP of the conjugates was determined according to the method described by Oyaizu et al. [40]. It is based on the reduction of ferricyanide [Fe(CN)_6_]^−3^ to the ferrocyanide [Fe(CN)_6_]^−4^ anion. Owing to this reduction, Prussian blue was formed in the presence of Fe^3+^. PBS (0.2 M, pH = 6.6; 50 μL) and 50 μL of K_3_[Fe(CN)_6_] (1%) were added to 20 μL of the samples at the concentrations of 15, 7.5, 3.75, and 1.88 mg/mL in one 96-well microplate. The mixture was incubated at 40 °C for 20 min. Subsequently, 50 μL of TCA (10%) and 10 μL of FeCl_3_ (0.1%) were added and incubated for 10 min at 40 °C. The absorbance was read at 700 nm using a microplate reader.

#### 3.8.2. Hydroxyl Radical Scavenging Activity

The percentage hydroxyl radical (OH·) scavenging capacity of the conjugates was determined according to the method developed by Lin et al. [41]. In this procedure, OH· is generated using the Fenton reaction. Thus, 20 μL of each sample at the concentrations of 15, 7.5, 3.75, and 1.88 mg/mL were placed in a 96-well microplate. Subsequently, 50 μL of 1,10-phenanthroline and FeSO_4_ (3 mM) were added. To start the reaction, 50 μL of H_2_O_2_ were added and incubated at 37 °C for 60 min. The absorbance was measured using a microplate reader at 536 nm.

### 3.9. Statistical Analysis

The statistical analysis of the data obtained during the determination of the extension of conjugation and the antioxidant activity tests was performed using GraphPad Prism v6.0, La Jolla, CA, USA. A one-way analysis of variance (ANOVA) and Tukey’s test were performed for multiple comparisons. The statistical significance was set at *p* ≤ 0.05.

## 4. Conclusions

The conjugation of peptide fractions from germinated soybeans to Fe_3_O_4_ NPs was achieved for the first time using a multicomponent protocol. The U-4C reaction was an efficient and versatile procedure for the conjugation of bioactive peptide fractions to Fe_3_O_4_ NPs. The experiments on the RP and hydroxyl radical scavenging activity demonstrated that the antioxidant capacity of the peptide fractions was maintained or increased after the conjugation. The only exception was the conjugation of the >10 kDa peptide fraction to the magnetite NPs coated with sodium citrate (conjugate 1). Its RP decreased slightly with respect to the nonconjugated peptide fraction. Fe_3_O_4_@APTES@ > 10 kDa showed the highest antioxidant capacity. The obtained results suggest a synergistic effect of the conjugation of peptide fractions from germinated soybeans to the coated Fe_3_O_4_ NPs by the U-4C reaction. 

## Figures and Tables

**Table 1:** Determination of the degree of substitution on the MNPs using the bicinchoninate method.

Particle	t (h)	Cu^2+^ Adsorption Capacity (mg Cu^2+^/g MNPs)	Amount (mmol of Free Groups/g MNPs)
Fe_3_O_4_@citrate	0	0	0
Fe_3_O_4_@citrate	1	14	0.44
Fe_3_O_4_@citrate	24	15	0.47
Fe_3_O_4_@APTES	0	0	0
Fe_3_O_4_@APTES	1	12	0.76
Fe_3_O_4_@APTES	24	12	0.76

## Data Availability

Data of this research is available from the authors.

## Sample Availability

Samples of the compounds Fe_3_O_4_@APTES@ > 10 kDa are available from the authors.
